# Bi-Hemispheric Engagement in the Retrieval of Autobiographical Episodes

**DOI:** 10.1155/2005/460745

**Published:** 2006-02-27

**Authors:** Marie M. P. Vandekerckhove, Hans J. Markowitsch, Markus Mertens, Friedrich G. Woermann

**Affiliations:** ^1^University of BielefeldPhysiological PsychologyP.O. Box 10 01 31D-33501 BielefeldGermany; ^2^Clinic for EpilepsyMaraBielefeld-BethelGermany

**Keywords:** Autobiographical episodic memory, emotion, neural correlates, fMRI

## Abstract

Functional magnetic resonance imaging (fMRI) was used to study the neural correlates of neutral, stressful, negative and positive autobiographical memories. The brain activity produced by these different kinds of episodic memory did not differ significantly, but a common pattern of activation for different kinds of autobiographical memory was revealed that included (1) largely bilateral portions of the medial and superior temporal lobes, hippocampus and parahippocampus, (2) portions of the ventral, medial, superior and dorsolateral prefrontal cortex, (3) the anterior and posterior cingulate, including the retrosplenial, cortex, (4) the parietal cortex, and (5) portions of the cerebellum. The brain regions that were mainly activated constituted an interactive network of temporal and prefrontal areas associated with structures of the extended limbic system. The main bilateral activations with left-sided preponderance probably reflected reactivation of complex semantic and episodic self-related information representations that included previously experienced contexts. In conclusion, the earlier view of a strict left versus right prefrontal laterality in the retrieval of semantic as opposed to episodic autobiographical memory, may have to be modified by considering contextual variables such as task demands and subject variables. Consequently, autobiographical memory integration should be viewed as based on distributed bi-hemispheric neural networks supporting multi-modal, emotionally coloured components of personal episodes.

## 1. Introduction

Episodic memory allows us to experience ourselves through the course of subjective time, from the past to the future, and to re-experience our previous life events [42]. Episodic memory is cognitive, symbolic and declarative [41]. As the main subset of episodic memory, autobiographical memory is directed towards the ecphory of past events. Tulving [42] considered ecphory as the process whereby retrieval cues interact with stored information so that an image or a representation of the information occurs. For the retrieval of episodic memories, however, a still controversial question is whether the right hemisphere contributes more intensely and more directly to the retrieval of affect-laden memories. The HERA (hemispheric encoding retrieval asymmetry) model of Tulving [44] and a number of more recent findings, both from functional neuroimaging [5] and from case reports of brain damaged patients [12,27], emphasise a stronger role of the right hemisphere. On the other hand, other authors have stressed a left-hemispheric [21,30], or a bilateral [25, 28,29,33] contribution to episodic memory retrieval. For the retrieval of episodic memories, a question is which is still undergoing active discussion is whether the right hemisphere contributes more intensely and more directly to the retrieval of affect-laden memories. More generally, it is not yet known how much overlap exists in brain activity produced by retrieving diverse kinds of autobiographical events [29].

The present study aimed at examining these unresolved issues by using memories of various valences, including positive, negative, stressful and neutral mem-

ISSN 0953-4180/05/$17.00 © 2005 – IOS Press and the authors. All rights reserved ories. Additionally, we required the participants to try to re-experience their autobiographical episodes intensely. These ranged from neutral to negative, positive and stressful memories. Stress, as we viewed it here, has been considered an important variable of influence, superimposed upon the positive or negative valence of an event. Examples of stressful (or pressured) situations include those situations with non-specific or specific time pressures, such as are experienced when submitting a manuscript in order to meet a deadline or when preparing for an important examination in mathematics, whereas examples of neutral situations include working in the garden, preparing dinner, or cleaning an apartment. Examples of negative situations include the death of one's grandmother or being abandoned by one's partner, and, finally, examples of positive situations include being accepted for a highly desired job or having a particularly beautiful evening with a friend.

Using functional magnetic resonance imaging (fMRI), the present study addressed the question of whether autobiographical memories of positive, negative, stressful, or neutral kinds recruit similar or different neural networks. A major question concerned, furthermore, the possibility of a differential recruitment of right versus left hemispheric networks during memory retrieval. The results obtained should have direct implications for the classification of memory tasks with respect to systems, laterality and processes.

## 2. Methods

### 2.1. Participants

Participants were 16 right-handed healthy women aged 21 to 32 years. None had a history of previous neurological or psychiatric illnesses or syndromes. No brain abnormalities were found on magnetic resonance scans. All were native German speakers. Informed consent was obtained from each prior to participation. Only females were used in order to limit possible gender effects [20,37].

### 2.2. Procedure

Prior to brain scanning, participants were informed that psychologists have studied memory for decades, but that there is still a need for research on the brain representation of autobiographical memory. On the morning of the day of the brain scanning, they were asked to retrieve neutral, stressful, negative and positive autobiographical episodes (see Introduction) evoked by a fixed set of six sentences per category (questions are given in Table 1). Participants were asked to rate and comment on the intensity of their emotion for each episode provided. In spite of obvious differences in content between individuals, all episodes were carefully selected with respect to their emotionality and their prompt evocation. All 24 cues (6 neutral, 6 stressful, 6 positive and 6 negative memories) were arranged in four blocks of six successive episodes of the same quality (neutral, stressful, positive and negative). Before the fMRI-measurements, participants practised using a computer programme to become familiar with the key words and the time-constraints of the procedure (extracted from their retrieved episodes by the psychologist conducting the experiment). This procedure corresponds to the one used before in several of our functional imaging studies [28,33] and allowed individuals to familiarise themselves with the procedure employed during scanning. For neutral- and stressful episodes, participants were encouraged to provide events as neutral and as stressful as possible. For negative and positive episodes, they were encouraged to provide events with an emotional degree of intensity and vividness as high as possible. Following an alternation or boxcar design, every retrieval period of 25 s alternated with a resting condition of the same length. The sequence of sessions was counterbalanced between the participants, rather then randomised, to avoid variable order artefacts. During brain scanning, participants had to re-imagine their original episodes after providing them with pairs of acoustically presented cue- (or trigger-) words. To control for episodic floating during the condition of rest, participants were instructed to imagine the Fig. 7 that the psychologist had shown before against a white background. Participants were instructed to keep their eyes closed throughout the whole time in the scanner. The questions depicted in Table 1 were used.

### 2.3. Post-scan debriefing

After scanning, participants were again asked to complete a questionnaire on the specific characteristics of their memories defined according to: 1. emotionality, 2. the experiential vividness of each episode, and 3. visual detail. These characteristics had to be rated on scales ranging between 1 = not at all, 2 = scarcely, 3 = slightly, 4 = fairly, 5 = much so, and 6 = very much so. Participants were paid for their participation.

Table 1
Questions used to trigger emotive episodes from the past

| |
|---|
| *1. Neutral memories* |
| 1. Gardening situation |
| 2. Shopping situation |
| 3. Lunch situation |
| 4. Working situation |
| 5. Cooking situation |
| 6. Cleaning situation |
| *2. Stressful memories* |
| 1. Stress situation |
| 2. Difficult examination |
| 3. Time pressure |
| 4. Important appointment |
| 5. Something lost |
| 6. Work stress |
| *3. Negative memories* |
| 1. Death of beloved person |
| 2. Sad situation |
| 3. Situation of loneliness |
| 4. Being hurt |
| 5. Situation of hopelessness |
| 6. Disappointment |
| *4. Positive memories* |
| 1. Being in love |
| 2. Happy moment |
| 3. Cheerful party |
| 4. Joyful situation |
| 5. Nice holiday |
| 6. Nice appointment |

### *2.4. MRI hardware and technical parameters*

MRI scans were carried out using a 1.5 T scanner (Siemens MAGNETOM Symphony, Erlangen, Germany) equipped with a standard head coil and with echoplanar imaging capability. To position the axial T2*-weighted images along the AC-PC line, scout and sagittal T1-weighted images were obtained in every participant. Functional imaging volumes were acquired according to a block paradigm, where a baseline condition was alternated with a stimulus condition. During each retrieval period, lasting 25 s, 10 sets of 16 axial T2*-weighted MR-slices with a slice thickness of 5 mm were obtained using a standard EPI sequence (TR = 1600 ms, TE = 50 ms, field of view [FOV] 192 mm, matrix 64 $\times$ 64). The volumes covered the upper part of the brain from vertex to the upper bank of the temporal lobes. To provide an anatomical reference and to exclude gross brain pathology, a T1-weighted 3 D-sequence (MPRAGE, TR = 11.08 ms, TE = 4.3 ms, slice thickness 1.5 mm, FOV 201 $\times$ 230 mm, matrix 224 $\times$ 256) was obtained in every participant.

All images were exported from the measurement console to a SUN workstation. Before intra-run realignment of the functional data sets, the first two images were skipped to account for T1 saturation effects during the first scans. The remaining images were analysed using SPM99, which was used for image pre-processing as well as for a voxel-based statistical analysis within the context of the General Linear Model (GLM). To correct for head movements, the images were realigned using the SPM99 default algorithm. Prior to group comparisons, anatomical differences were compensated for by means of a spatial normalisation using the SPM99 default settings and the standard stereotactic space, i.e. the MNI (Montreal Neurological Institute) brain. Then a spatial smoothing was done with a Gaussian kernel of 10 mm full-width-half-maximum (FWHM), to increase both signal and anatomical conformity.

### *2.5. Statistical analysis*

A fixed-effects statistical analysis for all permutations of the four conditions (neutral, positive, negative, stress) was done on a voxel-by-voxel basis. Differences between conditions were assessed individually as well as for the group of participants. After filtering (high-pass: 120 s, low-pass: hrf) t-statistic maps (height threshold: $t = 4.54$, $p = 0.001$ uncorrected) were generated for all contrasts and a single contrast image was generated from the parameter estimates for each contrast and for every participant. Then, with this single summary image per participant, a random effects analysis was conducted for the contrasts between each of the conditions using a one sample t-test model. A random effects analysis was also conducted for all memories (neutral, positive, negative and stressful) versus baseline. The resulting activation maps were superimposed to the MNI brain template of SPM99. Activations were projected onto representations of the standard stereotactic space as defined by Talairach and Tournoux [40] ("SPM 99 glass brains").

## 3. Results

### *3.1. Behavioural Results*

The results of the questionnaire concerned with the characteristics of the different types of autobiographical memory provided a significant effect of the conditions 1. Neutral, 2. Stressful, 3. Positive and 4. Negative autobiographical memory for all variables: emotionality [$F(3, 332) = 91.95, p < 0.001$ (df = amount of memories)], experiential vividness [$F(3, 329) = 7.097$,

$p < 0.001$], and visual detail [$F(3, 330) = 2.76$, $p < 0.05$]. Duncans post-hoc analysis was applied to detect whether the four kinds of autobiographical memory differed significantly ($p < 0.05$) from each other with respect to emotionality, vividness and amount of detail recalled. With respect to emotionality, negative memories were experienced as most emotional, positive and stressful were experienced as next most emotional, and neutral memories were experienced as least emotional. With respect to vividness, positive, negative and stressful memories were felt to be similarly vivid and all more so than neutral memories, which were felt to be least vivid. With respect to the amount of visual detail recalled, this was felt to be similar for neutral, negative and positive memories, all of which were associated with more recalled details than stressful memories, consistent with the view that stress may inhibit recall of details. Although recall of neutral episodes was felt to be less vivid, interestingly, participants recalled relatively large amounts of detail about these memories. The participants, therefore, reported that the four kinds of recalled episode had broadly the levels of emotionality, vividness and detail that one would expect.

### 3.2. *Neuroimaging results*

The four different types of autobiographical memory (neutral, stressful, negative and positive) were compared with each other and with the resting condition. Comparisons between the four kinds of autobiographical memory did not reveal any significant differences. However, a significant common pattern of brain activity was found across all conditions (relative to baseline), including largely bilateral portions of (1) the medial and superior temporal lobes, hippocampus and parahippocampus, portions of (2) the dorsolateral cortex and anterior medial and superior prefrontal cortex, (3) the anterior and posterior cingulate, including the retrosplenial cortex, (4) the parietal cortex, and portions of (5) the cerebellum (see Table 2 and Fig. 1).

## 4. Discussion

This study examined whether there were differences between the activations produced by recall of four kinds of autobiographical memories that varied with respect to the amount and kind of emotional experience with which they were associated (neutral, positive, negative and stressful), and it also examined what common activations were produced by recall of these different kinds of autobiographical experiences. Interestingly, no significant activation differences were produced by recall of these emotionally different kinds of autobiographical experience. Some recent literature suggests that emotion itself relies on distributed neural systems, with different emotions controlled by different (though overlapping) brain networks [10,28]. The lack of differences within the present study probably indicates a high degree of commonality in the brain mechanisms underlying diverse kinds of autobiographical memory, perhaps partially reflecting the self-referential character of all autobiographical memories. It is also possible that significant differences between the four conditions would have been obtained if more memories per condition or more participants had been used so as to increase power. The first option, however, was not considered feasible given the length of the task and the strong task demands that the participants already had to fulfil; the second option was unfortunately also not feasible because of time limits for the study. The behavioural results, which revealed significant differences between the conditions, showed, however, that it cannot plausibly be argued that the four conditions did not differ from each other with respect to their phenomenological characteristics. So, null results cannot be explained as a trivial result of a failure to get participants to generate episodes that differed emotionally. On the other hand, an important finding of this study is that the data obtained indicate that autobiographical memories, even when they differ in their emotional content, share many attributes, which suggests that retrieval of very different autobiographical memories depends on a common neural network. Activated areas correspond to the known networks supporting both non-autobiographical and autobiographical memory retrieval. The brain regions that were principally activated constituted an interactive network of structures that included (1) the medial and superior temporal lobes, the hippocampus, and the parahippocampal region, and (2) the anterior and posterior cingulate, including the retrosplenial cortex as well as (3) the dorsolateral cortex and anterior medial and superior prefrontal cortex, (4) the parietal cortex, and (5) the cerebellum.

The areas activated in the different conditions share a common medial temporal-prefrontal pattern and primarily comprise areas of the prefrontal cortex, the medial and lateral temporal cortex as well as the cingulate and retrosplenial cortices. Extensive neuroscientific evidence has indicated that prefrontal cortex and medial temporal lobe structures play important roles in

Table 2
Areas activated in the combined condition of neutral, positive, negative, stressful autobiographic memory relative to rest

| Region, Gyrus | BA | x | y | z | z-statistics |
|---|---|---|---|---|---|
| Inferior frontal gyrus | (47) | −36 | 25 | −6 | Inf |
| ,, ,, | (47) | −45 | 26 | −6 | Inf |
| ,, ,, | (47) | 53 | 29 | −7 | 6.17 |
| ,, ,, | (47) | 45 | 26 | −4 | 6.08 |
| Middle frontal gyrus/dorsol. g. | (10/46) | −39 | 45 | 20 | 4.61 |
| Superior frontal lobe | (9) | 15 | 57 | 28 | 5.55 |
| Middle temporal gyrus | (21) | −53 | −7 | −22 | 7.46 |
| ,, ,, | (21) | −48 | −38 | −8 | 5.96 |
| Parahippocampal gyrus | (28) | −18 | −18 | −17 | 6.54 |
| Hippocampus | | −27 | −13 | −17 | 5.29 |
| Superior anterior temporal g. | (38) | 48 | 16 | −28 | 6.10 |
| ,, ,, | (38) | −39 | 16 | −34 | 5.51 |
| Anterior cingulate gyrus | (32) | −6 | 20 | 42 | Inf |
| Posterior ,, ,, | (23) | −6 | −58 | 10 | Inf |
| Inferior parietal lobe, precuneus | (39) | −39 | −68 | 36 | Inf |
| ,, ,, | (39) | −48 | −63 | 23 | 6.14 |
| Posterior parietal lobe, precuneus | (7) | −15 | −70 | 49 | 5.65 |
| Cerebellum, posterior lobe | | 6 | −83 | −21 | 5.13 |

All these activations were significant at a threshold of $t = 4.54$ with an uncorrected $p < 0.001$.

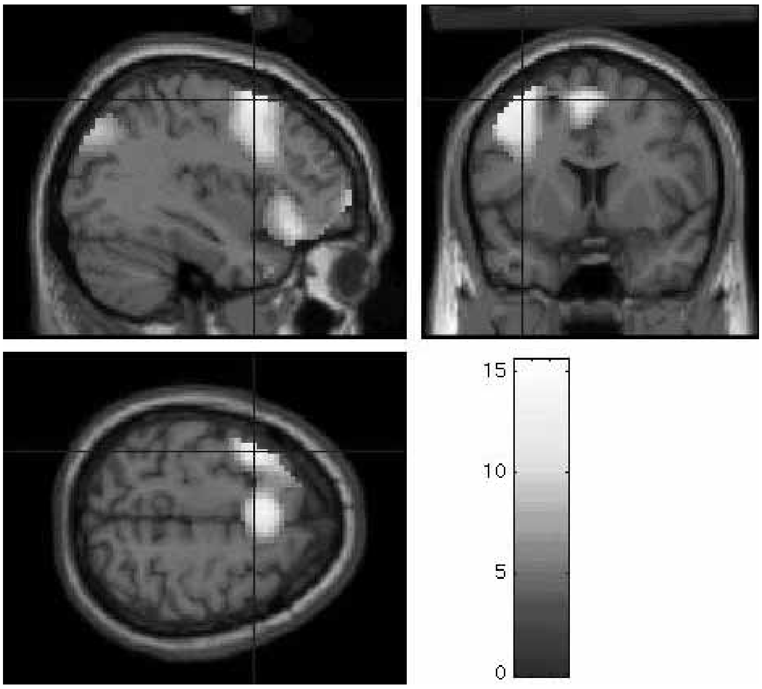

Fig. 1. Relative increases in neural activity associated with positive, negative, and stressful remembrances (compared to rest) superimposed on MRI sections to depict the functional anatomy of the activations and their relationship to the underlying structural anatomy.

recollective episodic memory function [15,38]. Graham et al. [16], for instance, compared the outcome of three positron-emission-tomographic studies of autobiographical memory and found a bilateral activation pattern in the middle temporal gyrus (BA 21) and medial frontal cortex (BA 9/10) (BA = Brodmann area). On the one hand, the bilaterally rather symmetrical activations correspond to the outcome of some previous research on episodic memory retrieval [3,26–28,33]. On the other hand, the activations seem to be partially in tension with the traditional HERA-concept and previous research by others [14,27]. The slightly different patterns of reported results have led to competing theories regarding the contributions of the left and right hemisphere and prefrontal regions to memory formation and retrieval. Taken together, the results of all these studies strongly suggest that laterality within the brain is more complex than previously suggested, especially with respect to autobiographical memory processing, and that the validity of existing models needs to be further verified, in order to further specify the role in episodic autobiographical memory of the left hemisphere in particular.

### *4.1. Prefrontal lobes*

The hypothesis that different prefrontal regions implement different functions with respect to long-term memory processing has been confirmed by our results with neuroimaging as well as by other previous workers [16,27,28,33]. Ventrolateral prefrontal lobe (BA 47) activation may principally be concerned with the active maintenance of retrieved information in working memory, as well as with the evaluation of the emotional significance of remembered events that have been selected within a context of associated competing memories [10,32]. Dorsolateral activations are probably correlates of monitoring, operating and verification processes that are applied when representations are retrieved [15,32]. Furthermore, self-referential processing – a central factor of autobiographical memory – has been associated with anterior medial-to-superior prefrontal activations within Brodmann area 10 (BA 10), and may account for our presently observed activations in this area [19].

The left prefrontal activation in our study corresponds to other recent results [8,30,33] and may refer to distinct stimulus characteristics and inherent processes (emotionality, perceptual detail, ease of retrieval). In a study of Ranganath et al. [34], for instance, activation of the left anterior prefrontal cortex (BA 10/46), increased with demands to retrieve perceptually detailed information on the items under study. Similarly, we had instructed our participants to retrieve perceptual and contextual details vividly and in detail. In addition, the complex demands of the retrieval task in our study may have enhanced both (1) the primary search and selection processes within the ecphory of an appropriate memory event and (2) the generation of cues for retrieving currently unactivated perceptual memory representations [9,18].

### *4.2. Parietal lobes*

Various aspects of mental imagery such as mentally moving through a spatial environment [2,13,31], including where and when an event took place, may explain the present activation of the parietal lobes during the recollection of autobiographical memory.

### *4.3. Temporal lobes*

Temporal lobe activation is consistent with several functional imaging studies on episodic memory [11, 17], and autobiographical memory in particular [14,28, 33]. Lateral and anterior temporal activity, especially in Brodmann areas 21 and 38, correspond to that observed in previous studies on lifelike events and autobiographical memory [6,14,25,26]. Within the temporal lobes, medial portions including the hippocampus and parahippocampus are particularly engaged in memory-related processes [11], including the successful ecphory of episodic events, their spatiotemporal context and their sensory-perceptual details [1]. Left temporal activity corresponds to that seen in a number of other episodic and autobiographical memory studies [25,26,28], and may stress the involvement of semantic factual knowledge while remembering an autobiographical memory.

### *4.4. Cingulate cortex*

Both anterior and posterior/retrosplenial cingulate activity was obtained corresponding to that seen in previous studies on memory retrieval [22,25,30]. A number of possible functions attributed to the (anterior) cingulate cortex can be assumed to explain the present activations, such as (1) helping to mediate attentional modulation so as to maintain the episodic memory retrieval mode and (2) re-experiencing emotions during recollection of an autobiographical event [22, 28]. The posterior cingulate/retrosplenial cortex has been described as important in processing and remembering of topographical information [24,39]. In a review, Maguire [24] has reported that topographical disorientation results in most cases with a retrosplenial lesion. Takahashi and colleagues [39] reported in three patients with right retrosplenial lesions a loss of the 'sense of direction' that allows one to recall the positional relationships between one's current location and one's intended destination within a space that cannot be entirely surveyed at one time.

### 4.5. Cerebellum

The role of the cerebellum in memory has proven highly intriguing. Cerebellar regions have recently been implicated in cognitive and emotional memory tasks [14,28,43]. The present activations may possibly reflect its participation in initiating and monitoring conscious retrieval of episodic memory and in updating the entire retrieval process [17].

## 5. Conclusions

The brain regions principally activated in various types of autobiographical memory investigated here overlapped considerably and constituted an interactive network of temporal and prefrontal areas defined as structures of the extended limbic system that connects affect and cognition. The main bilateral activations with left-sided preponderance probably reflect recall of complex, self-related information that includes a high level of detailed re-experiencing of multimodal contexts and perceptions. Considering the diverse conditions employed in this study, the primary search and the systematic self-cueing processes have to be seen as involving a detailed, deliberate analysis of activated information and the initiation of enhanced selection processes. Furthermore, due to its rich contextual nature, autobiographical memory contains both episodic (event-based) and semantic aspects. The contribution of both episodic and semantic systems can contribute to the actual outcome of autobiographical episodes in parallel and with different weights [21,28,29,40]. Autobiographical memory retrieval may benefit from recruiting the resources of both hemispheres. Consequently, a dynamic functional interaction between both left and right hemispheric functions is likely to underlie the semantic as well as the episodic processes that play a critical role in autobiographical memory retrieval. Different variables, such as task demands and associated operating and monitoring variables, subject variables and contexts, contribute dynamically and may activate both hemispheres to varying degrees [22]. Even allowing for possible methodological shortcomings of the present study, the overall pattern of common activations in different areas with different forms of autobiographical memory, stresses the importance of common distributed networks for such a complex task as autobiographical memory retrieval.
